# The cytochrome P450 inhibitor, ketoconazole, inhibits oxidized linoleic acid metabolite-mediated peripheral inflammatory pain

**DOI:** 10.1186/1744-8069-8-73

**Published:** 2012-09-24

**Authors:** Shivani Ruparel, Dustin Green, Paul Chen, Kenneth M Hargreaves

**Affiliations:** 1 Department of Endodontics, University of Texas Health Science Center at San Antonio, 7703 Floyd Curl Drive, San Antonio, TX, 78229, USA; 2 Department of Physiology, University of Texas Health Science Center at San Antonio, San Antonio, TX, USA

**Keywords:** Cytochrome P450, TRPV1, OLAMs, Inflammation

## Abstract

**Background:**

Oxidized linoleic acid metabolites (OLAMs) are a class of endogenous agonists to the transient receptor potential V1 (TRPV1) receptor. Although TRPV1 mediates inflammatory heat hyperalgesia, it is not known if the OLAMs contribute to the peripheral activation of this receptor during tissue inflammation. In the present study, we evaluated whether the OLAM system is activated during inflammation and whether cytochrome P450 enzymes mediate OLAM contributions to heat hyperalgesia using the complete Freund’s adjuvant (CFA) model of inflammation.

**Results:**

Our results demonstrate that the intraplantar (ipl) injection of anti-OLAM antibodies significantly reversed CFA-induced heat hyperalgesia. Moreover, application of lipid extracts from inflamed rat skin to cultured sensory neurons triggered a significant release of iCGRP that is blocked by co-treatment with I-RTX, a TRPV1 antagonist. To determine the role of CYP enzymes in mediating OLAM effects, we used a broad spectrum CYP inhibitor, ketoconazole. Pretreatment with ketoconazole inhibited the release of TRPV1 agonists in lipid extracts from inflamed skin and significantly reversed CFA-induced heat hyperalgesia by a peripheral mechanism of action. Moreover, the ipl injection of linoleic acid to rats 24 hr after CFA evoked spontaneous nocifensive behaviors that were significantly reduced by capsazepine, by knockout of the TRPV1 gene, or by pretreatment with either anti-OLAM antibodies or ketoconazole.

**Conclusions:**

Taken together, our data suggests that OLAMs contribute to inflammatory nociception in the periphery and that cytochrome P450 enzymes play a crucial role in mediating OLAM contributions to inflammatory heat hyperalgesia.

## Introduction

Tissue injury leads to the release of inflammatory mediators that sensitize and activate peripheral nociceptors resulting in an afferent barrage to dorsal horn neurons. The transient receptor potential vanilloid subtype-1 (TRPV1) receptor is a ligand-gated cation channel that is expressed on certain nociceptors, is activated by noxious stimuli such as heat, and mediates inflammatory heat hyperalgesia [1,2]. During tissue inflammation, TRPV1 activity is modulated by several classes of mediators such as arachidonic acid metabolites, bradykinin, nerve growth factor (NGF), as well as prostaglandins [3-5].

One class of endogenous mediators that activate TRPV1 consists of oxidized linoleic acid metabolites (OLAMs), namely, 9-and 13-hydroxy-10E,12Z-octadecadienoic acid (9-HODE and 13-HODE) as well their metabolites, 9-oxoODE and 13-oxoODE. Previous studies have reported that the OLAMs are synthesized under inflammatory conditions such as atherosclerosis and rheumatoid arthritis [6-8] as well as shown to be increased in serum of patients with chronic pancreatitis [9]. In addition, several groups have detected OLAMs in endothelial cells, macrophages and neutrophils [10,11], cell types activated during tissue inflammation. However, only recently have OLAMs been demonstrated to activate TRPV1 in both peripheral tissues and spinal dorsal horn [12,13]. Importantly, there is a gap in knowledge of this system since it is not known whether OLAMs contribute to activation of peripheral TRPV1 during tissue inflammation.

In the current study, we investigated the peripheral action of OLAMs in the rat complete Freund’s adjuvant (CFA) model of inflammation. Previously, we have shown that CYP inhibtors block linoleic acid activation of TRPV1 in cultured sensory neurons [14], suggesting that CYP isozymes mediate the formation of TRPV1-active linoleic acid metabolites. Accordingly, we conducted parallel *in vitro* and *in vivo* studies to determine whether peripheral CYPs in inflamed tissue mediate OLAM activation of TRPV1.

## Methods

### Animals

All protocols were approved by the Institutional Animal Care and Use Committee of the University of Texas Health Science Center at San Antonio. Male Sprague–Dawley rats (Charles River Laboratories, Inc., Wilmington, MA, USA) were used for all the studies except in one experiment where wild-type and TRPV1 knockout C57BL/6 mice (Jackson Laboratories) were used. Animals were housed for at least 7 days prior to the experiments.

### Drugs

Ketoconazole, iodo-resinferatoxin (I-RTX) and capsazepine (CPZ) were purchased from Tocris (Ellsville, Missouri, USA). The goat anti-9-HODE and anti-13-HODE antibodies were purchased from Oxford Biomedical Research (Rochester Hills, MI). As a control, a non-specific goat IgG antibody was purchased from Sigma Aldrich (St. Louis, MO). Linoleic acid (LA) was purchased from Cayman Chemicals (Ann Arbor, MI). Ketoconazole was diluted in 32% methylpyrrolidinone (MPL)/Hank’s balanced salt solution (HBSS) to make a stock of 18 mM and further diluted in HBSS on the day of each experiment. I-RTX stock (2 mM) was made in 100% ethanol that was further diluted in HBSS on the day of use. CPZ was diluted in 20% DMSO/80% mineral oil. The linoleic acid solution was dried under nitrogen gas to remove ethanol, and resuspended in HBSS immediately before the experiment. The pH of this solution was confirmed to be 7.4 to ensure no activation of TRPV1 by protons.

### TG cultures

For calcium imaging experiments, 1-day-old cultures of rat trigeminal ganglia (TG) were used. TG’s were dissected from normal rats and plated on poly-D-lysine/laminin coated glass cover slips (BD Biosciences, San Jose, CA, USA) and grown in the presence of 10% FBS and 100 ng/mL NGF (Harlan, Indianapolis, IN, USA) as described previously [15]. For determination of CGRP release, TGs from three rats were cultured as described [16] and plated on 24-well poly-d-lysine-coated plate yielding ~8000 cells per well. The media were replaced at the end of 24 h and then 48 h later. All the experiments were performed on day 5 of the neuronal cultures.

### Calcium imaging

Fluorescence imaging to measure calcium accumulations in sensory neurons was performed as described [15]. The TG cultures were incubated with the calcium-sensitive dye, Fura-2 AM (2 μm; Molecular Probes, Carlsbad, CA, USA) in Hanks modified buffer. Fluorescence was detected with a Nikon TE 2000U microscope fitted with a × 40/1.35 NA Fluor objective. Data were collected and analyzed with MetaFluor Software (Universal Imaging Corporation, Downingtown, PA, USA). The net changes in calcium influx were calculated by subtracting the intracellular calcium [Ca^2+^i level (mean value collected for 60 s prior to agonist addition) from the peak [Ca^2+^i value achieved after exposure to the agonist. Cells were pretreated with vehicle or ketoconazole (30uM) for 15 min followed by the addition of linoleic acid (1 mM) in the presence of the ketoconazole for 2 min. At the end of each experiment, a solution containing KCl (250 mM) was applied after linoleic acid application to ensure sampling of viable neurons. The ratio of 340/360 above 0.03 was considered to be a positive response for calcium influx.

### Model of inflammation

Male rats were anesthetized with isoflurane and injected with 50 μl of a 1:1 mixture of complete CFA (Sigma) in saline into right hind paw. At 24 hours, the inflamed tissue were either used to collect biopsy punches for lipid extract preparation or behavioral tests with inflamed hind paws.

### Preparation of lipid extracts

Rats were injected with CFA and at 20 hours, the animals were injected either with vehicle or ketoconazole (9.5 mg/kg) s.c. underneath the neck. Four hours after drug injection, the animals were decapitated and the inflamed hind paw tissues were collected by 6 mm biopsy punches. The tissues were diced and transferred to HBSS modified with CaCl_2_ 2 mM, NaHCO_3_ 4 mM, HEPES 10 mM, and dextrose 10 mM (pH 7.4) for 30 min at 37°C to wash and equilibrate. The tissues were then transferred to fresh HBSS buffer containing either vehicle or ketoconazole (150uM) and incubated at 37°C for 1 h. The superfusates were collected and subjected to lipid extraction using the C_18_-SepPak™ column (Waters, Inc) as described previously [12] and dried under a stream of N_2_. The dried samples were then resuspended in HBSS and applied to cultured sensory neurons for determining CGRP release.

### CGRP release assay and Radioimmunoassay

Calcitonin gene-related peptide (CGRP) release from TG neurons was determined as described previously [16]. Briefly, the experiments were conducted in HBSS solution using 5 day old TG cultures. After initial wash and baseline collection, the neurons were pretreated with either vehicle or I-RTX for 15 min prior to a 15 min exposure to the lipophilic extracts from inflamed skin. The media were collected and subjected to radioimmunoassay (RIA) for CGRP. All experiments were performed three times.

### Behavioral testing

All observers were blinded to treatment allocation. Thermal allodynia was measured using the radiant heat test as described previously [17]. To study the effects of HODE antibodies on CFA-induced heat hyperalgesia, 24 h inflamed rat hind paws were injected with 50ul of a mixture of anti-9-HODE (25 ug) and anti-13-HODE (25 ug) antibodies or 50ul of same amount of heat denatured antibodies. Thermal thresholds were measured 15, 30 and 60 min after injection. To study the effect of ketoconazole on CFA-induced heat hyperalgesia, vehicle or ketoconazole (4ug) was injected once 30 s prior to CFA injection in the ipsilateral hind paw and once 24 h later. Thermal thresholds were measured 1 h after the second ketoconazole injection. In a separate experiment, vehicle or ketoconazole (same dose) was injected in the contralateral paw 30 seconds before and 24 hours after CFA injection. Thermal hyperalgesia was measured 1 h later.

Nocifensive behavior was carried out as described previously [18] to study the effect of linoleic acid injection in inflamed rat hind paws. LA (10, 100 or 1,000 ug) or vehicle (50 uL) was injected ipl into normal and inflamed rat hindpaws and nocifensive behavior (defined as spontaneous flinching or flexion movements of the treated hind paw) was recorded every 2 min up to a total of 15 min. Similarly, LA-evoked nocifensive behavior was also tested in wild-type and TRPV1 KO mice. We then conducted three studies to determine the mechanism of LA-induced nocifensive behavior in rats after CFA inflammation. In all three studies rats were injected with CFA into the hind paw 24 h prior to the experiment. In the first study, rats were treated with capsazepine (50 mg/kg) or veh (sc), followed 1 hr later with LA injection. In the second study, rats were treated with a combination of anti-9- and anti-13-HODE antibodies (25 ug each) or non-specific goat IgG (ipl), followed 10 minutes later with LA injection. In the third study, rats were injected with ketoconazole (4ug) or veh (ipl) 30 minutes prior to LA injection.

### Statistics

Data are presented as mean ± SEM. Statistical analyses were performed using either a 2-tailed Student’s *t* test or one-way ANOVA with Neuman-Keul’s post-hoc test. A statistically significant difference was defined as *P* < 0.05. Error bars are S.E.M.

## Results

### OLAMs contribute to inflammatory heat hyperalgesia that is reduced by CYP inhibition

**Figure 1:**
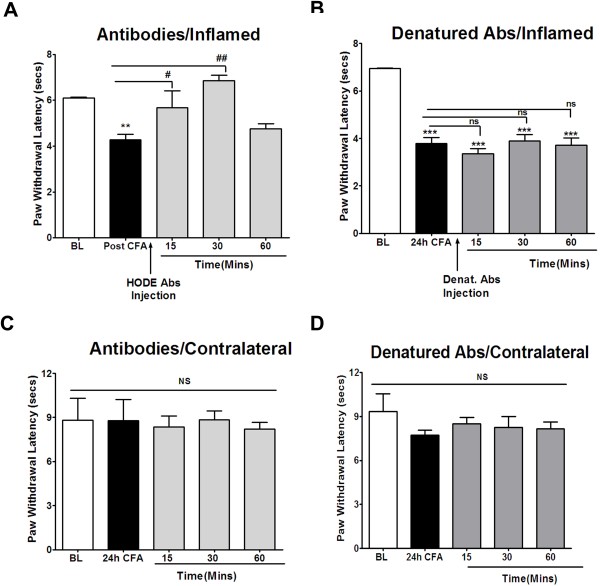
**Effect of HODE Antibodies on CFA-induced Thermal Allodynia.** A 50uL solution of 1:1 CFA:saline was injected into the right hind paw of rats and 24 h later, heat hyperalgesia was measured using the radiant heat test on the inflamed paws as well as in the contralateral (un-inflamed) paws. Following this, in one group of animals, 25ug of anti- 9-HODE and 25ug of anti-13-HODE antibodies were injected into the inflamed paw. Heat hyperalgesia was measured at 15, 30 and 60 minutes after antibody injection in the ***A.*** inflamed paw and ***B.*** in the contralateral paw. Similarly, another group of animals was injected with same amount of denatured HODE antibodies. Heat hyperalgesia was measured at 15, 30 and 60 minutes after antibody injection in the ***C*** inflamed paw and ***D.*** in the contralateral paw. n = 6 was used for each group in all the experiments and mean of all n’s is plotted. Data were analyzed using one-way ANOVA with Neuman-Keuls Post-hoc test. Error Bar: S.E.M.

To evaluate whether peripheral OLAMs contribute to inflammatory heat hyperalgesia, we injected a combination of anti-9-HODE and anti-13-HODE antibodies, or a mixture of the heat-denatured antibodies by the ipl route and blinded observers tested their effect on heat hyperalgesia. As expected (Figure 1A and 1B), CFA induced a significant heat hyperalgesia that was still evident at 24 h. Interestingly, injection of the HODE antibodies (Figure 1A), but not the heat denatured antibodies (Figure 1B), significantly reversed heat hyperalgesia for up to 30 min after ipl injection. Since this represented a complete reversal of heat hyperalgesia, the data are consistent with the hypothesis that peripheral OLAMs play a crucial role in inflammatory heat hyperalgesia. In contrast, there was no change in thermal latencies in the un-inflamed hindpaws that were contralateral to the hindpaws receiving either the mixture of antibodies (Figure 1C) or the denatured antibodies (Figure 1D).

**Figure 2:**
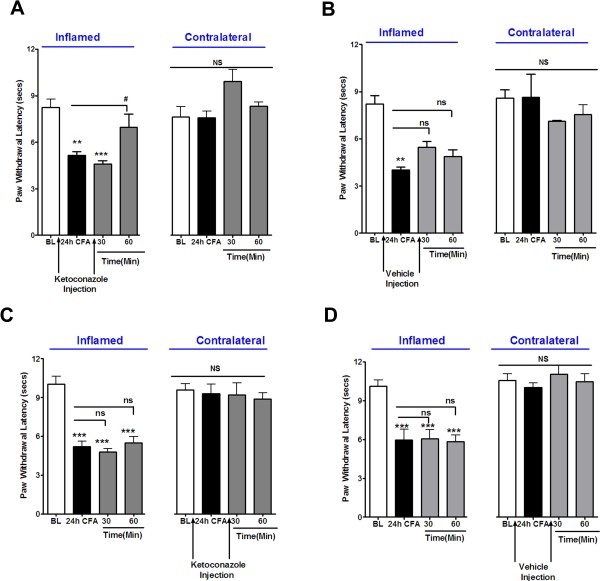
**Effect of Ketoconazole on CFA-induced Thermal Allodynia.** A 50uL solution of 1:1 CFA:saline was injected into the right hind paw of rats and 24 hours later, heat hyperalgesia was measured using the radiant heat test in the inflamed paws as well as in the contralateral paws. As noted, ketoconazole (4ug) was injected 30 sec prior to CFA injection and 24 hours later, thermal allodynia was measured in the ***A.*** inflamed paw and in the contralateral paw, following which another injection of ketoconazole (4ug) was injected in the inflamed paw. Heat hyperalgesia was tested at 30 and 60 minutes after second injection in the ***A*** inflamed paw and in the contralateral paw. In a separate group of animals, vehicle was injection 30 seconds prior to CFA injection. Heat hyperalgesia tested at 24 hours in the ***B.*** inflamed as well as contralateral paws, following which another vehicle injection was given and heat hyperalgesia tested at 30 and 60 minutes in the ***B.*** inflamed as well as in the contralateral paws. For data set ***C*** and ***D***, the experiment was performed in the similar fashion as 2A and B, except ***C.*** ketoconazole or ***D.*** vehicle was injected in the un-inflamed paw and heat hyperalgesia measured in ipsilateral paw (inflamed) as well as contralateral paw, 30 and 60 minutes after second injection. n = 6 was used for each group in all the experiments and mean of all n’s is plotted. Data were analyzed using one-way ANOVA with Neuman-Keuls Post-hoc test. Error Bar: S.E.M.

Next, we studied the role of CYP enzymes in regulating OLAM-mediated thermal hyperalgesia during inflammation. The ipl injection of ketoconazole into inflamed paws significantly reduced inflammatory heat hyperalgesia 1 h after drug injection compared to the vehicle injected group (Figure 2A and 2B). This effect is peripherally mediated since injection of ketoconazole into the contralateral hindpaws had no effect on the inflamed paws (Figure 2C “Inflamed” versus Figure 2D “Inflamed”). Moreover, the effect of ketoconazole was restricted to reversing heat hyperalgesia in inflamed hindpaws, with no changes observed in thermal antinociception in normal hindpaws, since local injection of ketoconazole into the un-inflamed hindpaws had no effect on paw withdrawal latencies (Figure 2C “Contralateral” versus Figure 2D “Contralateral).

### Ketoconazole inhibits the release of endogenous TRPV1 agonists

**Figure 3:**
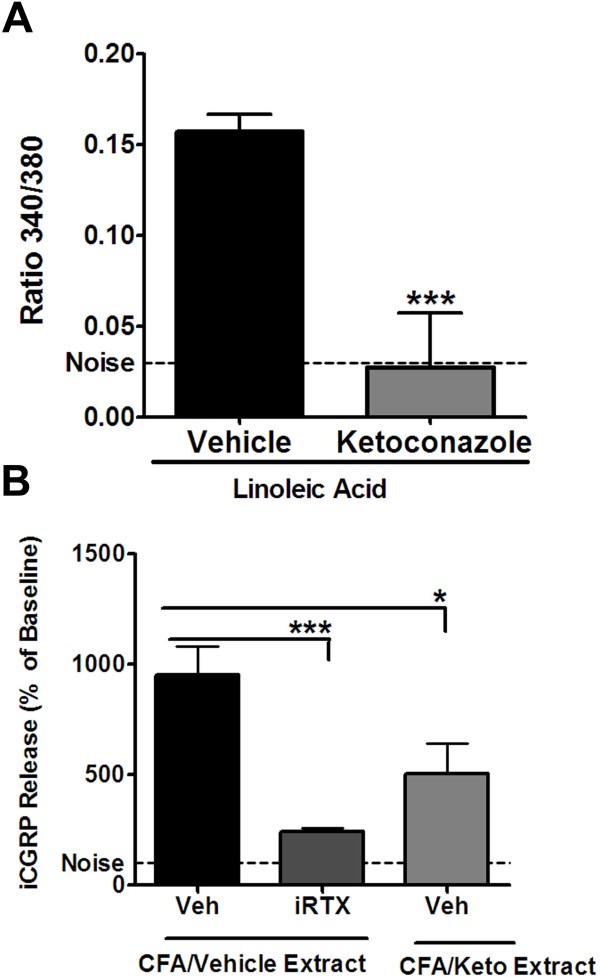
**Release of TRPV1 agonists.*A.*** Twenty-four hour old rat TG cultures were pretreated with either vehicle or 30uM ketoconazole for 15 minutes following which 1 mM LA with vehicle or the same concentration of ketoconazole was applied to the neurons for 2 minutes and calcium accumulated was tested by measuring ratio of fluorescence produced at 340 and 380 nm wavelengths. The experiment was performed with three independent neuronal cultures and mean responses for each group is plotted. Data were analyzed using 2-tailed student’s *t*-test. Error Bars: S.E.M. Noise indicates basal fluorescence ratio 340/360 with application of Hanks Buffer. ***B.*** Lipophilic extracts were made from inflamed skin from rats, pretreated with vehicle or 18 mM ketoconazole *in vivo* as well as vehicle or 150uM ketoconazole *ex vivo* and applied onto 5-day old rat TG cultures for 15 minutes. Neurons were pretreated with either vehicle or 1uM I-RTX for 15 minutes prior to extract application. The superfusates were collected and subjected to radioimmunoassay for estimation of CGRP release. Three independent experiments were performed and data plotted is the mean of % release from baseline for each group. Data were normalized such that baseline release from neurons was made to be 100%. Data were analyzed using one-way ANOVA and Neuman-Keuls post hoc test. Error Bars: S.E.M.

We previously have reported that LA-evoked calcium accumulation in sensory neurons is TRPV1-dependent and is blocked by compounds that inhibit cytochrome P450 enzyme activity, such as carbon monoxide, nordihydroguaiaretic acid and diphenyliodonium [14]. However, several of these compounds are not suitable for *in vivo* studies and so we evaluated the well known CYP inhibitor, ketoconazole. Cultured TG neurons that were pretreated with vehicle followed by stimulation with LA exhibited a three-fold increase in [Ca]i compared to noise (Figure 3A). In contrast, pretreatment with ketoconazole abolished the effect of LA on triggering increased [Ca]i (p < 0.005). Thus, ketoconazole inhibits the formation of TRPV1-active metabolites of linoleic acid.

We next evaluated whether ketoconazole alters the release of endogenous TRPV1 agonists from inflamed tissues (Figure 3B). At 24 hr after injection of CFA, inflamed tissue biopsies were collected and lipids were extracted and isolated using reverse phase chromatography. The application of the resuspended extracts to cultured TG neurons evoked a ten-fold increase in CGRP release that was significantly attenuated in extracts co-treated with the TRPV1 antagonist, I-RTX. These data support the release of endogenous TRPV1 agonists during CFA inflammation. To determine if these substances were released by oxidative enzymes, a separate group of animals received *in vivo* administration of ketoconazole prior to biopsy collection with continued exposure to ketoconazole during the *ex vivo* extraction process. The resuspended extracts were then applied onto TG cultures with a significant, although incomplete, inhibition of CGRP release. Taken together, these data indicate endogenous TRPV1 agonists are released during CFA inflammation and that about one-half of the activity can be attributed to a ketoconazole-sensitive mechanism.

### Linoleic acid-evoked nocifensive behavior during inflammation is TRPV1 dependent

**Figure 4:**
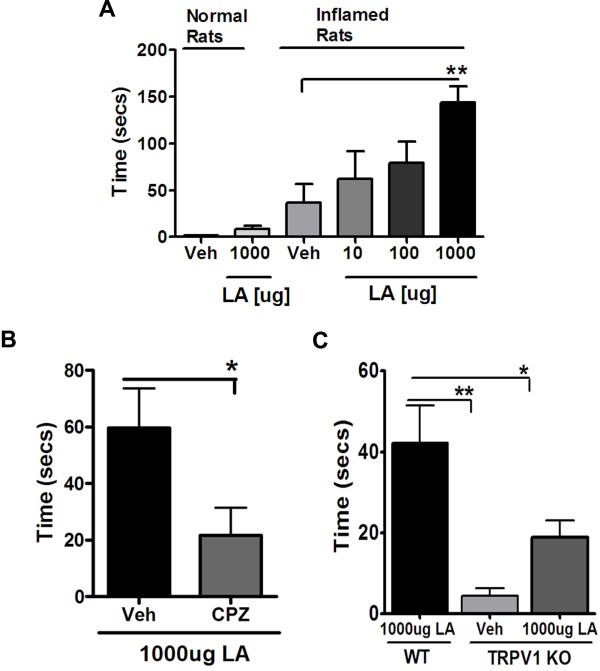
**Effect of Linoleic acid on Nocifensive Behavior in Rats and Mice.*A.*** Rats were injected either with saline or 1:1 CFA and 24 hours later, vehicle, 10, 100 or 1000ug linoleic acid was injected into normal uninflamed and/or inflamed hindpaws and nocifensive behavior was recorded upto 15 minutes. Data are plotted as total nocifensive behavior time (in seconds) within 15 minutes. n = 6 was used for each group. Data Analyzed using one-way ANOVA with Neuman-Keuls post hoc test. Error Bar: S.E.M. ***B.*** Rats were injected with 1:1 CFA and 24 hours later, 50 mg/kg capsazepine or vehicle was injected systemically underneath the neck. 1 hour later, 1000ug of LA was injected in the inflamed paws and nocifensive behavior was recorded upto 15 minutes. Data plotted is the total nocifensive time (in secs) observed between 8 and 15 minutes. n = 8 was used for each group. Data analyzed using 2-tailed Student’s *t*-test. Error Bar: S.E.M. ***C.*** Wild-type mice or TRPV1 KO mice were injected with 1:1 CFA and 24 hours later 1000ug LA was injected in the inflamed paws of wild-type and TRPV1 KO mice. Nocifensive behavior was recorded and plotted between 8 and 15 minutes after injection. A vehicle group was included in the TRPV1 KO mice as well. Data plotted is the total nocifensive time (in sec) observed between 8 and 15 minutes. n = 8 was used for each group. Data Analyzed using one-way ANOVA with Neuman-Keuls post hoc test. Error Bar: S.E.M.

If the OLAM system is active during CFA inflammation, then administration of exogenous LA to inflamed hindpaws would be predicted to trigger nocifensive behaviors. In control (un-inflamed) hindpaws, the ipl injection of LA did not produce a significant increase in spontaneous nocifensive behaviors (Figure 4A). However, at 24 h after CFA inflammation was established, the ipl injection of LA produced a dose-dependent nocifensive effect (Figure 4A). This suggests that the machinery responsible for OLAM synthesis is minimally active under control conditions, and is substantially upregulated following tissue inflammation. We next evaluated whether LA-induced nocifensive behaviors in inflamed hindpaws was mediated by activation of TRPV1. In rats, pretreatment with capsazepine significantly reduced LA-evoked nocifensive behaviors (Figure 4B). Similarly, LA-triggered nocifensive behaviors were significantly reduced in mice with genetic deletion of TRPV1 as compared to wild type mice (Figure 4C). Thus, inflammation enhances LA-induced nocifensive behaviors and these behaviors are due, at least in part, to TRPV1 activity.

**Figure 5:**
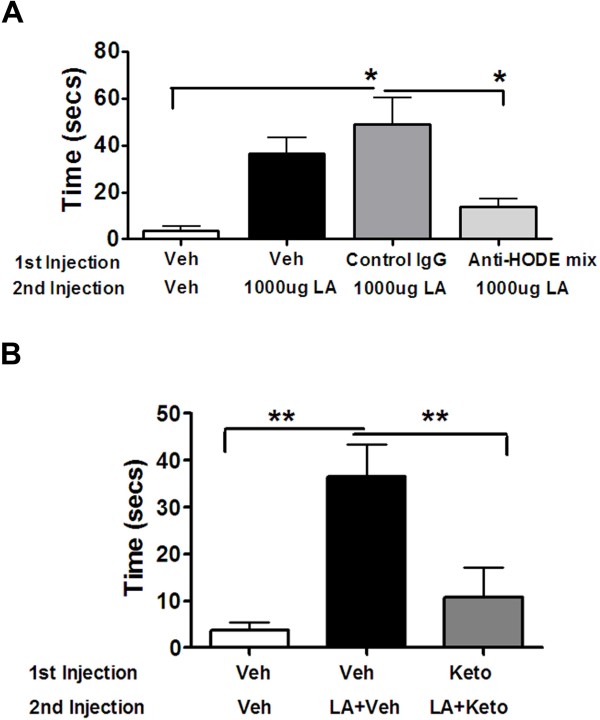
**Effect of anti-HODE Antibodies and Ketoconazole on LA-evoked Nocifensive Behavior.*A.*** Rats were injected with 1:1CFA and 24 hours later, were injected with either vehicle, control IgG anti-goat antibody or mix of anti-9 and 13-HODE antibodies (25ug each). Following 10 minutes, a second injection of either vehicle or 1000ug LA was given and nocifensive behavior was recorded upto 15 mins. Data plotted is the total nocifensive time (secs) observed between 8 and 15 mins. n = 8/10 was used for each group. Data analyzed using one-way ANOVA with Neuman-Keuls post hoc test. Error Bar: S.E.M. ***B.*** Similar to data set 4A, rats were injected with vehicle or 4ug ketoconazole as the first injection into the inflamed paws and 30 minutes later, vehicle or 1000ug LA with vehicle or 1000ug LA with 4ug ketoconazole was given as the second injection. Data plotted is the total nocifensive time (secs) observed between 8 and 15 mins. n = 8/10 was used for each group. Data Analyzed using one-way ANOVA with Neuman-Keuls post hoc test. Error Bar: S.E.M.

Since LA can be metabolized to various different polyunsaturated fatty acids (PUFAs) [19-23], we noted in our preliminary studies the nocifensive effects of LA *in vivo*, produced a TRPV1 dependent response between 8 and 15 minutes after LA injection. Hence in subsequent experiments, we focused on this time range and therefore the data shown in Figure 4B, 4C and Figure 5 is nocifensive behavior observed only between 8 and 15 minutes after LA injection.

### Anti-HODE antibodies and Ketoconazole inhibit linoleic acid-induced nocifensive behavior

We next evaluated whether administration of exogenous LA triggers nocifensive behaviors via enzymatic formation of OLAMs. As seen in Figure 5A, the ipl injection of a combination of anti-9 and anti-13-HODE antibodies reduced LA-induced nocifensive behavior by ~72% compared to the group that received either the control anti-goat IgG antibody or the vehicle (HBSS) group.

Next, to further study the role of CYP enzymes in OLAM synthesis, we pretreated the inflamed rat hindpaws with ketoconazole 30 minutes prior to LA injection and observed that ketoconazole also inhibited LA effect by ~70% (Figure 5B).

## Discussion

Oxidized metabolites of linoleic acid are elevated in inflammatory conditions such as atherosclerosis and rheumatoid arthritis and chronic pancreatitis [6-9]. Recently, our group has demonstrated that some of these metabolites, namely, 9-HODE, 13-HODE and their oxo-metabolites, are endogenous TRPV1 agonists [12,13]. The present study has expanded the knowledge of the OLAMs by implicating their role in evoking inflammatory heat hyperalgesia. Thus, OLAMs may not only mediate acute responses to thermal stimulation in sensory neurons [12,13], but may also participate in more prolonged inflammatory conditions.

Several independent lines of evidence were pursued to test this hypothesis. First, peripheral injection of anti-HODE antibodies were shown to reverse inflammatory heat hyperalgesia. Second, lipid extracts of inflamed hind paw skin were shown to contain endogenous TRPV1 agonists. Third, ketoconazole, a broadly acting inhibitor of oxidative enzymes, was demonstrated to inhibit the release of endogenous TRPV1 agonists from inflamed skin, to inhibit CFA-induced heat hyperalgesia and to inhibit the effects of exogenous linoleic acid for triggering spontaneous nocifensive behaviors. Collectively, these data provide strong support for the hypothesis that OLAMs contribute to inflammatory heat hyperalgesia.

An additional finding in this study is that exogenous linoleic acid triggers spontaneous nocifensive behaviors when injected into inflamed, but not control hind paw tissue. This effect of linoleic acid was dose-related and was due, in part, to activation of TRPV1 as indicated by the use of capsazepine in rats and studies conducted in TRPV1^-/-^ mice. However, the data also reveal an additional non-TRPV1 mechanism for linoleic acid-evoked nocifensive behavior The effects of linoleic acid are significantly reduced by pretreatment with anti-9- and anti-13-HODE antibodies, implicating these mediators in contributing to spontaneous nocifensive behaviors. Interestingly, this immunoneutralization experiment did not completely block the nocifensive effects of linoleic acid. This incomplete blockade might be due to the formation of non-HODE OLAMs (e.g., 9-oxoODE or 13-oxoODE), to a non-OLAM mechanism, or to insufficient amount of antibodies (i.e., 1,000 ug of linoleic acid is ~3.5 umol versus ~0.3 nmol antibody). It is not likely that the “non-OLAM” mechanism is due to protons, since the linoleic acid solution and the vehicle were both buffered to a pH of 7.4 prior to injection. Since linoleic acid can be a substrate for the formation of epoxy-9Z-octadecenoic acids, the corresponding dihydroxy acids, as well as arachidonic acid and its metabolites, several potential non-OLAM mediators are possible [19-21,23,24]. For example, arachidonic acid metabolites such as 12- and 15-(*S*)-hydroperoxyeicosatetraenoic acids, 5- and 15-(*S*)-hydroxyeicosatetraenoic acids as well as leukotriene B4 are class of polyunsaturated fatty acids that are capable of activating TRPV1 [4,5] It is notable that inflammation substantially upregulates the machinery necessary for linoleic acid to evoke spontaneous nocifensive behavior. One potential mechanism is upregulation of oxidative enzymes capable of metabolizing linoleic acid. This possibility is consistent with the observation that ketoconazole significantly reduces linoleic acid-evoked nocifensive behaviors. Indeed, microarray studies have reported upregulation of several CYPs after CFA injection [25], including CYP3A1, an enzyme expressed in about 40% of TRPV1^+^ neurons [14]. However, the results do not exclude the possibility that the enhanced LA response could also be in part due to changes in TRPV1 expression, trafficking or phosphorylation.

This study also found that ketoconazole has an unexpected antihyperalgesic effect. This action of ketoconazole is peripherally mediated since injection into contralateral hindpaws had no effect. The effect appears to be due, at least in part, to inhibition of the OLAM system, since ketoconazole blocked linoleic acid-induced elevation in [Ca]i in cultured neurons and significantly reduced linoleic acid-induced nocifensive behaviors in rats. Moreover, treatment with ketoconazole inhibited the release of endogenous TRPV1 agonists from inflamed tissue. However, since ketoconazole inhibits multiple oxidative enzymes including CYPs, the biochemical mechanism of action requires future evaluation. Taken together, the finding that ketoconazole only acts in the injured area, suggests that it may serve as a prototype for a novel class of peripherally active analgesics.

Collectively, the current study implicates a role of OLAMs in inducing inflammatory heat hyperalgesia in the periphery and suggests that oxidative enzymes such as cytochrome P450 isozymes play an important role in OLAM synthesis. It would be worthwhile to study the cell types in the skin tissue that are predominantly active in the OLAM-CYP system. For example, several CYPs and other oxidative enzymes are expressed in immune cells [26,27] and we and others have demonstrated the expression of several CYP isoforms in sensory neurons, including the subpopulation that express TRPV1 [14,28,29]. Hence it is possible that OLAMs are synthesized in the immune cells or neuronal terminals. The finding that anti-OLAM antibodies reduced CFA-induced heat hyperalgesia and linoleic acid-evoked nocifensive behaviors strongly support a paracrine function of the OLAMs during tissue inflammation since it is not likely that the antibodies cross plasma membranes. Identification of the cell type expressing these enzymes and the specific isoforms driving OLAM synthesis may yield potential targets for novel analgesic drug development.

## Competing interests

The authors declare they have no competing interests.

## Authors’ contributions

Dr. SR and Dr. KH designed all experiments. SR carried out in vitro experiments with calcium imaging, lipid extract preparation and CGRP radioimmunoassays as well as drafted the manuscript. Dr. KH edited the manuscript as well as supervised the entire study. DG and PC performed behavioral experiments. All authors read and approved the final manuscript.
